# Development of gut inflammation in mice colonized with mucosa-associated bacteria from patients with ulcerative colitis

**DOI:** 10.1186/s13099-015-0080-2

**Published:** 2015-12-21

**Authors:** Zhengyu Du, Tomas Hudcovic, Jakub Mrazek, Hana Kozakova, Dagmar Srutkova, Martin Schwarzer, Helena Tlaskalova-Hogenova, Martin Kostovcik, Miloslav Kverka

**Affiliations:** Institute of Microbiology, The Czech Academy of Sciences, Prague, Czech Republic; Institute of Microbiology, The Czech Academy of Sciences, Nový Hrádek, Czech Republic; Institute of Animal Physiology and Genetics, The Czech Academy of Sciences, Prague, Czech Republic; Institute of Experimental Medicine, The Czech Academy of Sciences, Prague, Czech Republic

**Keywords:** Dysbiosis, Microbiota, Germ-free mice, Ulcerative colitis

## Abstract

**Background:**

Disturbances in the intestinal microbial community (i.e. dysbiosis) or presence of the microbes with deleterious effects on colonic mucosa has been linked to the pathogenesis of inflammatory bowel diseases. However the role of microbiota in induction and progression of ulcerative colitis (UC) has not yet been fully elucidated.

**Methods:**

Three lines of human microbiota-associated (HMA) mice were established by gavage of colon biopsy from three patients with active UC. The shift in microbial community during its transferring from humans to mice was analyzed by next-generation sequencing using Illumina MiSeq sequencer. Spontaneous or dextran sulfate sodium (DSS)-induced colitis and microbiota composition profiling in germ-free mice and HMA mice over 3–4 generations were assessed to decipher the features of the distinctive and crucial events occurring during microbial colonization and animal reproduction.

**Results:**

None of the HMA mice developed colitis spontaneously. When treated with DSS, mice in F4 generation of one line of colonized mice (aHMA) developed colitis. Compared to the DSS-resistant earlier generations of aHMA mice, the F4 generation have increased abundance of *Clostridium difficile* and decrease abundance of *C. symbiosum* in their cecum contents measured by denaturing gradient gel electrophoresis and DNA sequencing.

**Conclusion:**

In our study, mucosa-associated microbes of UC patients were not able to induce spontaneous colitis in gnotobiotic BALB/c mice but they were able to increase the susceptibility to DSS-induced colitis, once the potentially deleterious microbes found a suitable niche.

**Electronic supplementary material:**

The online version of this article (doi:10.1186/s13099-015-0080-2) contains supplementary material, which is available to authorized users.

## Background

Crohn’s disease (CD) and ulcerative colitis (UC), the two major types of inflammatory bowel disease (IBD), are characterized by chronic relapsing inflammation of the gastrointestinal tract. This inflammation is a result of an aberrant immune response to antigens of resident gut microbiota [1, 2]. In spite of intensive research, however, the underlying mechanisms are still not fully elucidated. It has been proposed that either imbalances in intestinal microbiota (dysbiosis) or presence of commensal bacteria with increased virulence could both cause excessive immune response to microbiota by penetrating through the mucosal barrier and stimulating local and systemic immunity [3–5].

The gut microbiota ecology in UC patients is significantly different from the microbiota in healthy subjects, with typical reduction of diversity among major anaerobic species [6–8]. Transfer of this luminal dysbiotic microbial community to the germ-free mice renders them more susceptible to experimentally-induced intestinal inflammation than similar transfer from healthy subjects [9]. Due to the close contact with gut mucosa, the adherent microbes may be even more important for disease development. Dominant species of mucosa-associated bacteria are significantly different from those found in feces [10], and patients with UC have more bacteria attached to the epithelial surfaces than healthy individuals [11, 12]. But whether these alterations are cause or a result of the intestinal inflammation is still not entirely clear.

To study these mechanisms and to uncover the participation of bacteria in the development of inflammatory diseases, microbiota analysis is not sufficient and gnotobiology has to be employed. In contrast to established human disease, host-microbe interactions during early stages of the disease development can be studied by using animal models of inflammatory diseases in gnotobiotic conditions (i.e. in germ-free or artificially colonized animals with known microbes). In our previous experiments, acute intestinal inflammation induced by dextran sulfate sodium (DSS) was milder in germ-free (GF) mice compared to normally colonized mice [13], and the mode and timing of the colonization with microbiota modified the future immune phenotype of the host [14].

Since the composition and metabolic activities of intestinal microbiota of experimental animals are different from that of human gut microbiota [15], microbes relevant to the human disease could be missed by using animal models. To overcome this issue, GF animals can be colonized with human microbiota. These humanized or human microbiota-associated (HMA) animals are capable of maintaining the bacterial community of the human gut, thus keeping microbiota composition and its metabolic activities similar to those of the human intestine [16, 17]. Therefore, gnotobiotic animals can be used to cultivate bacteria that are uncultivable by most conventional methods [18].

In this study, three lines of HMA mice were created by colonization of the GF mice with bacteria present in colonic biopsies from three patients with active UC. Our aim was to test whether the mucosa-associated bacteria derived from UC patients could induce spontaneous colitis or render the mice more sensitive to DSS-induced colitis. The composition of bacterial community in cecum content of HMA mice was monitored for several generations to understand its dynamics with respect to colonization at adult age (parental generation) or neonatal mother-to-offspring (filial generations) mode of colonization.

## Results

### The inter-individual variability in biopsy samples

To measure the inter-individual differences among biopsy lysates, we estimated the beta diversity metrics using unweighted (qualitative) and weighted (quantitative) UniFrac. This qualitative analysis showed that biopsy b is significantly different from biopsy a and biopsy c and biopsy c was not significantly different from biopsy a. However, there were no differences among samples in quantitative analysis of beta diversity (Table 1). This suggests that abundances of major bacterial taxa are similar among all three biopsy samples and low abundance species contributed to the difference between biopsy b and biopsy a or biopsy c.

**Table 1:** Comparison of microbiota composition in biopsies

Sample 1	Sample 2	P	P (Bonferroni corrected)
Unweighted UniFrac			
Biopsy c	Biopsy a	0.06	0.90
Biopsy c	Biopsy b	0.00*	≤0.01*
Biopsy a	Biopsy b	0.00*	≤0.01*
Weighted UniFrac			
Biopsy c	Biopsy a	0.93	1.00
Biopsy c	Biopsy b	0.42	1.00
Biopsy a	Biopsy b	0.70	1.00

Biopsies show significant inter-individual differences only in presence of low abundance taxa as showed by qualitative (unweighted UniFrac) but not abundance-aware (weighted UniFrac) quantitative analysis. Statistically significant results are marked with asterisk

### The diversity of microbiota is decreased after the colonization

**Fig. 1:**
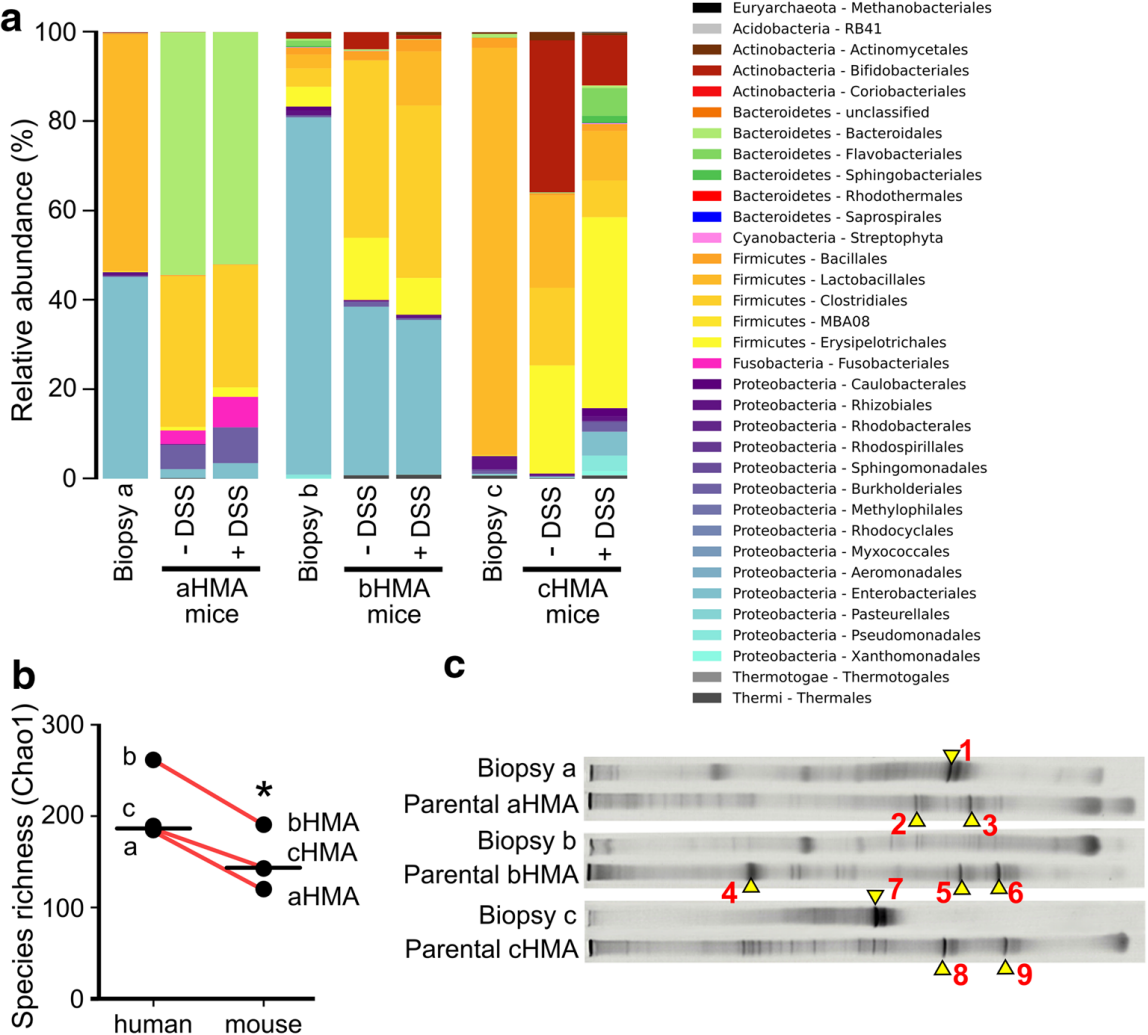
Microbiota composition in colonic biopsies of three patients with active ulcerative colitis and cecum content of parental HMA mice, **a** as measured by 16S sequencing. The composition of each sample is based on the RDP taxonomic assignment of the 16S rDNA sequences. The phylum and the genus level are shown for the most abundant bacterial groups. **b** The Chao1 diversity index of human biopsy samples (*a*, *b* and *c*) was compared with the diversity index of cecum contents of relevant mice (healthy aHMA, bHMA and cHMA) by two-tailed paired Student’s *t* test. The black line represent median and the *red lines* connect the related samples. **c** DGGE profiles of 16 s rRNA genes amplified from colonic biopsy lysates and cecum content of biopsy-colonized mice. Excised and successfully sequenced bands are identified with red numbers (*1*–*9*), see Table 1 for identification

GF mice were successfully colonized with bacteria from biopsies of three patients with active UC (Fig. 1a, c). Microbial community in samples from human biopsies is characterized by dominance of one or two bacterial orders, Lactobacillales and Enterobacteriales, which comprise more than 80 % of identified reads from each community. After the transfer of microbiota into the mice, composition of communities was shifted, with decrease in abundance of Lactobacillales compensated with an increase in other Firmicutes, namely with Clostridiales. Moreover, in general abundance distribution of bacterial orders in communities after the transplant was more evenly distributed but total species richness decreased during transfer from humans to mice (Fig. 1b). This decrease may be caused either by partial unsuitability of recipient niche for the bacterial community found in the biopsy samples or by dying of less abundant species during the transfer from human to mice. The presence and viability of multiple anaerobic and aerobic bacteria in biopsies and cecum of parental generation of HMA was confirmed by cultivation-based methods (Table 2) [19–21].

**Table 2:** Bacteria in the parental HMA mice and biopsy lysates used for their colonization, as identified by enzymatic tests and microscopy

	Aerobic bacteria	Anaerobic bacteria
Biopsy a	*Klebsiella oxytoca* *Proteus vulgaris* *Streptococcus parvulus*	*Actinomyces naeslundii* *Fusobacterium necrogenes/mortiferum*
P aHMA mice	*Enterococcus faecalis* *Enterococcus rafinosus* *Enterococcus faecium* *Streptococcus parvulus* *Klebsiella pneumonie* *Escherichia coli* *Proteus vulgaris*	*Veillonella parvula* *Bifidobacterium breve* *Bifidobaterium sp.* *Bacteroides capillosus* *Actinomyces israelli* Unidentified G + cocci-rods Unidentified G + rods
Biopsy b	*Escherichia coli* *Klebsiella pneumonie* *Enterococcus flavescens* *Enterococcus casseliflavus*	*Actinomyces naeslundii* *Veillonella parvula* *Bifidobacterium* sp.
P bHMA mice	*Enterococcus casseliflavus* *Enterococcus faecalis* *Enterococcus* sp. *Klebsiella pneumonie* *Citrobacter amalonaticus* *Escherichia coli*	*Veillonella parvula* *Eubacterium lentum* *Bifidobacterium sp.* *Actinomyces israelli* *Lactobacillus* sp.
Biopsy c	*Streptococcus* sp. *Enterococcus faecium* *Enterococcus raffinossus*	*Lactobacillus jensenii*
P cHMA mice	Yeast *Enteococcus faecium* *Enterococcus raffinossus* *Streptococcus parvulus*	*Clostridium inocuum* *Bifidobacterium* sp. Unidentified G + spore-forming rods

### Colonization of GF mice with mucosa-associated bacteria from IBD patients does not lead to spontaneous colitis

**Fig. 2:**
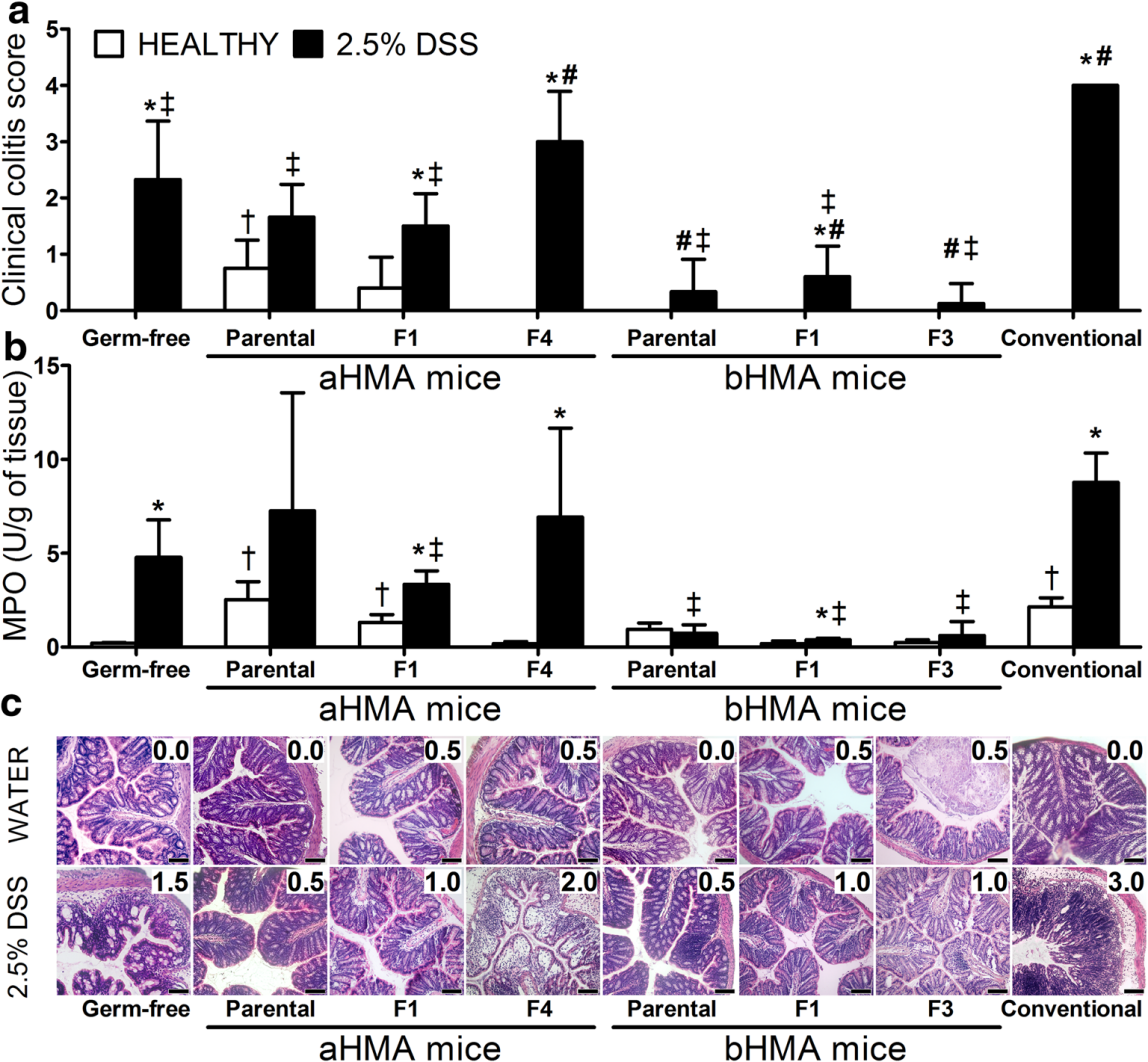
Macro- and microscopic evaluation of DSS induced colitis, as measured by **a** clinical colitis score, **b** colonic MPO activity, and **c** histological analysis of the mucosal damage of the colon descendens. The values are expressed as mean (*bar*) value ± standard deviation (*whisker*). *Each bar* represents 4– mice and histology (paraffin-embedded sections stained with haematoxylin and eosin) is from one mouse showing changes typical for each group. The numbers represent histological grade and the *black bar* is 100 µm. *p ≤ 0.05 vs. non-treated littermates; ^†^p ≤ 0.05, vs. healthy GF mice; ^#^p ≤ 0.05, vs. DSS-treated GF mice; ^‡^p ≤ 0.05, vs. DSS-treated CV mice; *F1/3/4* 1st/3rd/4th filial generation, *DSS* dextran-sodium sulfate

To test if bacteria from the UC biopsies can induce gut inflammation, each mouse was evaluated for colitis. Compared to water-treated GF mice, which remained completely healthy, the clinical colitis score (CCS) and myeloperoxidase (MPO) were significantly higher in water-treated parental and first filial generation (F1) of mice colonized with biopsy a (aHMA) (Fig. 2). Increase in MPO was also detected in water-treated parental bHMA mice, but no histological signs of colitis were observed in any group of water-treated HMA mice (Fig. 2c). Similar results were found in parental cHMA mice, which did not developed colitis either spontaneously or after DSS-treatment. The cHMA line of mice did not breed beyond parental generation and died out. This suggests that the mucosa-associated microbiota from patients with active UC cannot induce spontaneous colitis in mice, although the process of artificial colonization may induces slight inflammation of colonic mucosa.

### aHMA mice exhibited an increase in DSS-colitis sensitivity whereas bHMA mice failed to develop colitis

When treated with DSS, GF mice developed milder colitis than conventional (CV) mice, suggesting that the presence of microbiota increased the susceptibility of mice to colitis. There was a significant increase in CCS and MPO in GF, CV, F1 aHMA, F4 aHMA and F3 bHMA after DSS treatment, compared to their littermates treated with water (Fig. 2a, b). Compared to F1 aHMA mice, CCS and MPO value were higher in F4 aHMA mice though the differences in MPO were not statistically significant. The typical histopathologic picture of DSS-induced colitis was observed only in GF mice (mild to medium), F4 aHMA mice (moderate) and CV mice (very severe) with a characteristic massive loss of goblet cells and crypts, ulceration and inflammatory infiltrate in the lamina propria and submucosa (Fig. 2c). The increase in macro- and microscopic signs of colitis in aHMA mice shows an increase in DSS-colitis sensitivity over the generations. Interestingly both MPO values and CCS in water-treated aHMA mice showed a steady decline tendency over generations. In contrast to aHMA mice, bHMA mice failed to develop colitis in all groups of mice throughout the generations (Fig. 2a–c).

### Production of proinflammatory and regulatory cytokines is increased in colitic F4 aHMA mice

**Fig. 3:**
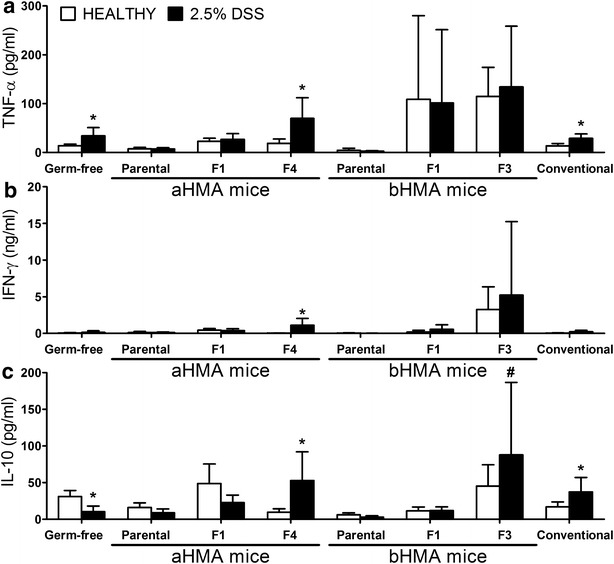
Cytokine production by spleen cells from HMA mice. Each group contained 4–8 mice. **a** TNF-α, **b** IFN-γ, and **c** IL-10 cytokine levels were measured in supernatant from spleen cells. Cytokine values are expressed as mean ± standard deviation, *p ≤ 0.05 vs. non-treated littermates; ^#^p ≤ 0.05, vs. DSS-treated GF mice

The production of proinflammatory cytokines Tumor necrosis factor (TNF)-α and Interferon (IFN)-γ in spleen cell suspension was higher in DSS-treated F4 aHMA mice than that in their healthy littermates (Fig. 3a, b). Higher production of TNF-α was also found in DSS-treated GF or conventional mice, but the levels of IFN-γ were not changed. Interleukin (IL)-10, known to regulate immune responses [22], was significantly higher in DSS-treated F4 aHMA mice when compared to their water-treated littermates (Fig. 3c). Interestingly, contrary to F4 mice, production of IL-10 was significantly higher in water-treated GF mice than in DSS-treated mice. On the other hand, no significant differences in cytokines production were determined between DSS-treated and water-treated bHMA mice. The differences in cytokine production between DSS-treated and untreated mice are, therefore, only apparent in mice with clear phenotype of DSS-induced colitis, such as DSS-treated GF, F4 aHMA and CV mice. These results suggest that changes in the cytokine pattern reflect more the presence of colitis in DSS-treated animals than the differences in microbiota that colonize the mice.

### HMA mice in the later generation exhibited higher biodiversity in intestinal bacterial community

**Fig. 4:**
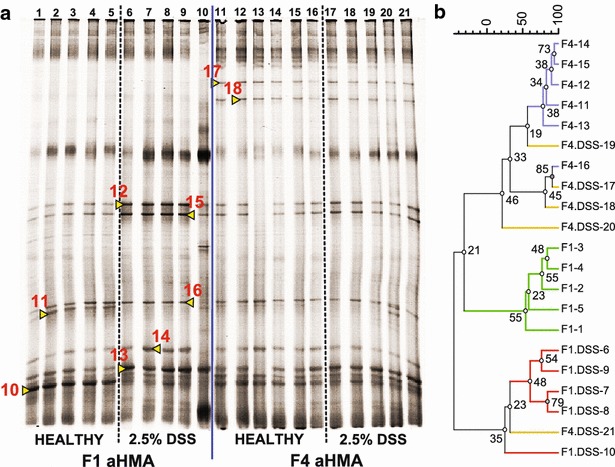
The differences in cecum microbiota of F1 and F4 aHMA mice. **a** DGGE profiles of 16 s rRNA genes amplified from cecum content of human biopsy A-associated mice. Each lane (*1*–*21*) represents a DNA sample isolated from cecum content of one mouse. Excised and successfully sequenced bands are identified with *red numbers* (*8*–*16*), see Table 1 for identification. **b** Clustering analysis of DGGE banding profiles of cecum samples. The dendrogram was generated by using the Wards method from a Pearson correlation matrix. The *numbers on the nodes* indicate the bootstrap values expressed as percentage from 1000 replications

**Fig. 5:**
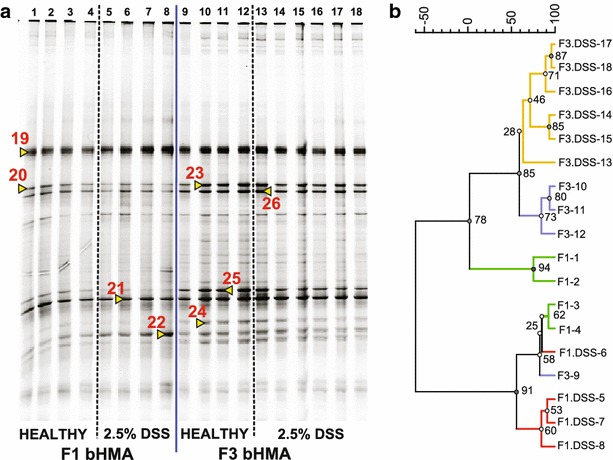
The differences in cecum microbiota of F1 and F3 bHMA mice. **a** DGGE profiles of 16 s rRNA genes amplified from cecum content of human biopsy B-associated mice. Each lane (*1*–*18*) represents a DNA sample isolated from cecum content of one mouse. Excised and successfully sequenced bands are identified with numbers (*17*–*24*), see Table 1 for identification. **b** Clustering analysis of DGGE banding profiles of cecum samples. The dendrogram was generated by using the Wards method from a Pearson correlation matrix. The *numbers on the nodes* indicate the bootstrap values expressed as percentage from 1000 replications

Shannon-Wiener index was used to compare the diversity of microbiota in cecum samples from HMA mice. Significantly higher diversity was measured in F4 aHMA mice and F3 bHMA mice compared to their previous generations (aHMA: F1 = 1.03 ± 0.03 vs F4 = 1.18 ± 0.06, p < 0.05; bHMA: F1 = 1.12 ± 0.03 vs F3 = 1.35 ± 0.09, p < 0.05). Interestingly, there was no significant difference in diversity between water-treated and DSS-treated HMA mice. Four clusters were roughly generated in cecum samples in each line of HMA mice and the samples from the same generation and treatment clustered well together (Figs. 4b, 5b).

### A predominance of colitis-associated *Clostridium sp*. was identified in cecum samples of aHMA mice but not in bHMA mice

Prominent bands from DGGE profiles (Figs. 4, 5) of PCR amplified DNA from cecum content (Additional file 1) were identified as *Clostridium sp*. and *Blautia sp*. in both aHMA and bHMA mice (Table 3). Compared with bHMA mice, in which DSS-induced colitis was not established, aHMA mice conserved higher richness of *Clostridium* species in their cecum samples (Table 1). Substantial amount of *C. difficile* and *C. aurantibutyricum* were identified in F4 aHMA mice, in which DSS-colitis was successfully developed. These mice have substantially lower abundance of *C. symbiosum* compared to healthy F1 aHMA (Fig. 4a), suggesting that this microbe has not been successfully transferred to the later generation of aHMA mice.

**Table 3:** Phylogenetic affiliation of DNA sequences retrieved from DGGE bands

Sample	No	GenBank Accession number	Best match	Identity (% similarity)
Biopsy a	1	NR121743	*Streptococcus lutetiensis*	99
P aHMA	2	NR118699	*Clostridium innocuum*	97
		NR044648	*Eubacterium tortuosum*	91
		NR113409	*Eubacterium dolichum*	90
P aHMA	3	NR119035	*Clostridium sphenoides*	99
P bHMA	4	NR118729	*Clostridium oroticum*	98
P bHMA	5	NR041960	*Blautia luti*	98
P bHMA	6	NR036800	*Ruminococcus gnavus*	100
Biopsy c	7	NC017960	*Enterococcus faecium*	98
P cHMA	8	NR119085	*Clostridium polysaccharolyticum*	97
P cHMA	9	AB971793	*Clostridium innocuum*	98
F1 aHMA	10	NR044715	*Clostridium clostridioforme*	96
		NR036928	*Clostridium hathewayi*	96
		NR118730	*Clostridium symbiosum*	96
F1 aHMA	11	NR118730	*Clostridium symbiosum*	99
F1 aHMA	12	NR119217	*Blautia producta*	99
F1 aHMA	13	NR041960	*Blautia luti*	100
F1 aHMA	14	NR118729	*Clostridium oroticum*	97
		NR117142	*Eubacterium fissicatena*	97
		NR104803	*Eubacterium contortum*	97
F1 aHMA	15	NR118729	*Clostridium oroticum*	96
		NR117147	*Eubacterium contortum*	96
		NR117142	*Eubacterium fissicatena*	96
F4 aHMA	16		No siginificant similarity found	
F4 aHMA	17	NR074454	*Clostridium difficile*	99
F4 aHMA	18	NR044841	*Clostridium aurantibutyricum*	98
F1 bHMA	19	NR118729	*Clostridium oroticum*	98
F1 bHMA	20	NR041960	*Blautia luti*	98
F1 bHMA	21	NR041960	*Blautia luti*	100
F1 bHMA	22	NR036800	*Ruminococcus gnavus*	100
F3 bHMA	23	NR118729	*Clostridium oroticum*	99
F3 bHMA	24		No siginificant similarity found	
F3 bHMA	25	NR118729	*Clostridium oroticum*	98
F3 bHMA	26	NR118729	*Clostridium oroticum*	99

If the identity of the best match was 97 % or less, two other matches were selected. The sequence number correspond to these in Figs. 1, 4 and 5. (For sequences refer to Additional file 1)

## Discussion

Inflammation in patients with UC is usually confined to large intestine, characterized by dysbiosis [23]. When transferred to GF mice, this dysbiotic microbial community in UC patients increase susceptibility to DSS-induced colitis [9]. Luminal microbes forming feces have often only indirect contact with inflamed colon mucosa, so mucosa-associated bacteria are more likely to be involved in UC due to their close proximity to the host epithelium. In healthy individuals, gut bacteria are usually separated from the intestinal mucosa by thick layers of mucus [24], thus even methods as sensitive as quantitative (q) PCR or Fluorescence in situ hybridization (FISH) is not able to detect any bacteria in most biopsies from healthy subjects [11, 25].

In this study, we found that major bacterial taxa are similar among all three biopsy samples we used for colonization and only low abundance species differ among biopsies from UC patients (Table 1). When the microbial community is transferred from human biopsies to GF mice, the species richness of this community is significantly reduced (Fig. 1b). This may be caused either by partial unsuitability of recipient niche for the bacterial community or by dying of less abundant species during the transfer from human to mice. This methodical difficulty could not be fully excluded even when freshly collected biopsies were used and their contact with oxygen in the air was minimized.

Colonization of GF mice with mucosa-associated microbiota from UC patient a (aHMA mice) increased CCS and MPO activity without damage to colon mucosa. CCS and MPO gradually decreased in subsequent generations, which support the notion that lack of exposure to microorganism in the early life could interfere with the development of immune system and permanently alter important immune functions [14]. Therefore, the increase in MPO and presence of pasty stool in parental aHMA mice appears to be a result of the poorly regulated host-microbe interaction in the ex-GF mice. The absence of mucosal damage in healthy HMA mice suggests that the mucosa-associated microbes from patients with active UC do not induce colitis when transferred to otherwise healthy host. However, this effect cannot be fully excluded, e.g. if some rare and strongly damaging microbial communities are transferred, due to the low number of individual biopsies we tested.

To investigate how the mucosa-associated bacteria increase the sensitivity to colitis, DSS-colitis was induced in GF, HMA and CV mice. Colitis was successfully induced in GF, F4 aHMA and CV mice with varying severity; mild-moderate in GF mice, moderate in F4 aHMA mice and very severe in CV mice. This is in agreement with our previous study showing that GF mice are more resistant to acute DSS-induced colitis than CV mice [13]. The presence of mild colon inflammation in GF mice suggests that microbiota is not indispensable for colitis development in this model. The absence of colitis in DSS-treated parental, F1 aHMA, F1 bHMA and F3 bHMA mice clearly shows that microbiota might contain certain protective species that actively protected mice from intestinal inflammation. Their presence would explain the failure to induction of DSS-colitis in all bHMA mice and in parental and F1 aHMA mice. The increase in susceptibility to DSS-induced colitis between F1 and F4 aHMA mice suggests that these protective bacteria may be lost or that other, potentially harmful microbes found suitable niche during natural colonization with co-housing. The differences in colitis sensitivity between both lines and different generations also show, that certain specific microbes, and not the presence of any microbe, is the cause of colitis sensitivity in F4 aHMA mice. We did not transfer the microbiota from healthy subjects because we expected that these biopsies will yield inoculums too low for successful colonization with complex microbiota [11, 25]. On the other hand, we cannot exclude that similar effects will be observed also with mucosa-associated bacteria from healthy subjects.

When we sequenced the bands that were different between F1 and F4 aHMA, we found disappearance of *C. Symbiosum* and appearance of *C. difficile. C. symbiosum* (member of the *Clostridium* cluster XIVa) is the most abundant bacterium found in human gut mucins, where it probably protects the mucosa by producing high levels of butyrate [26]. This effect may be responsible for the resistance of the F1 aHMA mice to the DSS-induced colitis. Disappearance of *C. symbiosum* during DSS treatment of F1 aHMA mice could be even partially responsible for the DSS-induced epithelium damage. *C. difficile*, on the other hand, may produce toxins that can damage colon mucosa of infected patients [27]. Indeed, there is a strong association between UC and colonization with this bacterium [19, 20], and this association is not limited only to toxin-producing *C. difficile* [23]. The close association of *C. difficile* with colitis may be responsible for the marked increase in susceptibility to DSS-colitis between F1 and F4 generations. Since all these microbes could not be introduced in other way than with the original biopsy, their appearance on DGGE of F4 aHMA suggests that they found suitable niche and increased in numbers. PCR-DGGE can detect only more dominant species, because its detection limit is between 10^4^ and 10^8^ cfu/ml, depending on the selected bacterium [28–30]. Reduced richness of intestinal microbiota is a common feature in UC patients [7, 31–34]. It is interesting that there is no significant reduction in biodiversity of microbiota in cecum samples of DSS-treated HMA mice compared to their healthy littermates. Taking into account that the biopsies were taken from patients with active UC, we can speculate that the bacteria transferred to mice were well adapted to inflammatory environment.

Intestinal inflammation is associated with impaired barrier function, which leads to activation of the systemic immunity and production of pro-inflammatory cytokines [35, 36]. In fact, this activation is less pronounced in the mucosal compartment, including mesenteric lymph nodes, than in systemic one, due to more active inhibitory mechanisms in the gut [37]. This effect is probably caused by the regulatory mechanisms of the mucosal immune system [38]. IL-10 is an important anti-inflammatory cytokine that regulate the colonic inflammation during experimental colitis in the presence of microbiota [39, 40]. Therefore, an increased IL-10 production in DSS-treated F4 aHMA mice and DSS-treated CV mice maybe caused by negative-feedback loop, where immune system regulates the inflammation caused by gut barrier breach. The observed decrease in IL-10 production in DSS-treated GF mice may be caused by the immunological immaturity, indicating that the GF mice do not have fully developed regulatory mechanisms on a level of innate and adaptive immunity [41, 42].

## Conclusions

In summary, we showed that mucosa-associated bacteria from colonic biopsy of the patients with active UC can increase sensitivity to DSS-induced colitis, although not able to induce spontaneous one. The increase in DSS-induced colitis severity between earlier and later generations of aHMA, together with the appearance of *C. difficile* and disappearance of *C symbiosum*, suggests that change in the relationship between these two particular microbes, rather than their presence or absence, is important for the sensitivity to colitis. Production of these “humanized” mice using patient’s biopsy and following the fate of bacteria over generations may bring new insights into host-microbe interaction during intestinal inflammation or in other diseases.

## Methods

### Patients and biopsy

Biopsy was taken from inflamed sites of colon descendens from three patients during routine endoscopic examination. First patient (a) was 52-year old male, diagnosed with active UC with shortened colon, caused by a chronic inflammation. Second patient (b) was a 23-year old male, diagnosed with very active UC resistant to both mesalasine (5-acetylosalycilic acid) and azathioprine treatment. Third patient (c) was 28-year old female, diagnosed with very active UC with numerous ulcers. Immediately after extraction, the biopsies were transferred to the laboratory in sterile tubes pre-loaded with Schaedler anaerobe broth (Oxoid Ltd, Cambridge, UK) containing 0.05 % cysteine-HCl, 10 % glycerol and covered with the layer of paraffin to preserve anaerobes.

### Animals

GF BALB/c mice (8–10 week-old) were maintained in isolators under sterile conditions, supplied with sterile water and sterile pellet diet ST-1 (Velaz, Unetice, Czech Republic) ad libitum, to keep them free of live bacteria. The conventional (CV) BALB/c mice on the same diet were regularly checked for the absence of potential pathogens according to an internationally established standard (FELASA).

### Human biopsy administration and experimental design

Each human biopsy was homogenized with sterile hand homogenizer, and 2-month old GF mice were colonized with 0.2 ml of this homogenate in a single gavage, which were employed as Parental HMA mice. All biopsies were processed immediately after the transport to the laboratory and under anaerobic conditions until the gavage. Three months later, parental HMA mice were divided into three groups; one group was continuing in breeding for reproduction; one group was used for colitis induction and the other was used as control against colitis induced mice. The offsprings of the parental HMA mice (F1) and the third (F3) and fourth (F4) generations were again divided into three groups as the parental HMA mice did. The microbiota composition of the biopsy homogenate and cecum content of the parental HMA mice (after 3-month colonization of biopsy homogenate) were analyzed by microscopic and cultivation methods (see Additional file 1: Table S1) and by next-generation sequencing (Fig. 1a, b) to analyze the microbiota viability and changes in microbiota diversity during the transfer from humans to mice. Colitis was induced in GF, HMA and CV mice by 7 days lasting exposure to 2.5 % (weight/volume) dextran sulfate sodium (DSS; Mw = 36–50 kDa; ICN Biomedicals, Cleveland, OH, USA) in sterile drinking water similarly, as described earlier [13, 43]. Controls received sterile drinking water. 8-week old mice were used in all experiments except Parental HMA mice. During the whole duration of these experiments, each line of HMA mice was kept in separate isolator to avoid any contamination with other microbes. The cHMA line of mice did not breed well and it died out shortly after the experiment with parental generation.

### Microbiota analysis by cultivation analysis and microscopy

The presence of live microbes in the biopsy lysate and in cecum content of the colonized mice was analyzed by cultivation-dependent methods with subsequent microscopic and enzymatic tests. Before plating, the whole cecum of colonized mice was removed and gently vortex in 5 ml of Schaedler broth containing 0.05 % cysteine-HCl. The samples were cultivated either aerobically using, bovine Blood agar, MRS agar with or without 0.05 % cysteine, Sabouraud agar (all from Oxoid, Hampshire, UK), Endo agar (Merc, Darmstadt, Germany) or anaerobically on VL blood agar (Imuna-Pharm, Slovak Republic). Next, the individual colonies were separated, cultivated and analyzed by microscopy after Gram’s staining and by detection of their oxidase (PLIVA-Lachema Diagnostika, Brno, Czech Republic) and katalase activity. Subsequently, their enzymatic activity was determined by oxidative-fermentative test, enterotest, anaerotest, en-coccustest or PYRtest (all from PLIVA-Lachema Diagnostika, Brno, Czech Republic). The software TNW^®^ (PLIVA-Lachema Diagnostika, Brno, Czech Republic) was used to identify the individual species of bacteria (Table 2).

### Evaluation of acute colitis

Each mouse was examined on day 8 for stool consistency (solid 0 points, loose stool that do not stick to the anus 2 points, and 4 points for liquid stools that stick to the anus) and rectal bleeding (none 0, positive guaiacum reaction 2 points, and 4 points for gross bleeding), and the clinical colitis score (CCS) was determined as a mean of these two parameters.

The colon was removed and its distal third was fixed in Carnoy’s solution for 30 min, and then transferred into 96 % ethanol, embedded in paraffin, sectioned at 5 μm transversal sections and stained with haematoxylin and eosin. Histological grade, ranging from normal (0) through borderline (0.5) to extreme colitis (3), was calculated by evaluating the degree of epithelium ulceration and infiltration of inflammatory cells in each colon segment according to a standardized histological scoring system [43].

### Myeloperoxidase (MPO) measurement

The extent of neutrophil infiltration was quantified by measuring MPO activity in the colon tissue homogenate, as described earlier by Krawisz et al. [44] with some modifications. Briefly, 1–2 cm of colon descendens (approximately 50 mg of tissue) was washed in ice-cold phosphate-buffered saline (PBS) and homogenized in 1 ml of potassium buffer (0.05 M KH_2_PO_4_, 0.05 M K_2_HPO_4_, pH = 6.0). After centrifugation at 12,000*g* for 30 min 4 °C, the pellet was resuspended in 1 ml 0.5 % hexadecyltrimethylammoniumbromide (HETAB) in 50 mM potassium buffer (pH = 6.0). Next, samples were sonificated for 30 s, freeze-thawed three times, sonificated again and centrifuged at 12,000*g* for 30 min. The supernatant was used for the measurement of the MPO activity. All steps of MPO extraction were carried out on ice.

MPO activity was measured by incubating 100 μl of the sample with 2.9 ml prewarmed 50 mM phosphate buffer (pH = 6.0) containing 16.7 % (wt/vol) o-dianisidine and 0.0006 % H_2_O_2_ at 37 °C. The reaction kinetics was measured at OD 460 nm for 3 min at 30 s intervals. The MPO activity is expressed in units (U) per 1 gram of the tissue, where 1 U equals the change of OD_460_ of 1 in 1 min.

### Measurement of cytokine production

Single-cell suspensions of spleens were prepared by mashing the tissue and passing the cells through the 70 µm sterile cell strainers (Becton–Dickinson, San Jose, CA, USA). After the lysis of red blood cells with sterile ACK lysing buffer (0.1 mM EDTA, 150 mM NH_4_Cl, 10 mM KHCO_3_), and two washes in complete culture medium (RPMI 1640 supplemented with 10 % heat inactivated FCS, 2 mM-glutamine, 100 U/ml penicillin, 100 mg/ml streptomycin), the cells were seeded at 5 × 10^6^ cells/500 µl of complete medium per well in 48-well flat bottom plates (Corning; Tewksbury, MA, USA) and cultivated for 48 h at 37 °C, 5 % CO_2_ in humidified incubator. The cell supernatant was then used for determination of IFN-γ, TNF-α and IL-10. The cytokines were measured by ELISA kit (R&D Systems; Mineapolis, MN, USA).

### DNA isolation

Bacterial DNA was isolated from cecum contents of HMA mice using ZR fecal Kit™ (Zymo Research, Irvine, CA, USA), according to the manufacturer’s protocol. The concentration and quality of isolated DNA was assessed by measuring its absorbance at 260 and 280 nm using spectrophotometer (NanoDrop Technologies, Inc; Wilmington, DE, USA) and its concentration was adjusted to 10 ng/μl.

### Polymerase chain reaction (PCR) and denaturing gradient gel electrophoresis (DGGE)

The sequences of bacterial 16S rRNA genes were amplified in the DNA isolated from cecum contents of HMA mice using the universal bacterial primers 338GC and RP534 in a previously described protocol for PCR assays (5′-CGC CCG CCG CGC CCC GCG CCC GGC CCG CCG CCG CCG CCG CAC TCC TAC GGG AGG CAG CAG-3′) and RP534 (5′-ATT ACC GCG GCT GCT GG-3′) in a previously described protocol for PCR assays [45]. Each PCR mixture contained 2 μl of DNA template, 0.5 μl of each primer (10 μM), 15 μl of ReadyMix™ Taq PCR Reaction Mix (Sigma-Aldrich, Steinheim, Germany), and 12 μl of nuclease-free H_2_O. Samples were initially denatured at 94 °C for 3 min, followed by 36 cycles of 1 min at 94 °C, 20 s at 61 °C and 40 s at 68 °C with final elongation at 68 °C for 7 min. Products from PCR were then processed by DGGE using the DCode™ Universal Mutation Detection System (Bio-Rad Laboratories, Hercules, CA, USA) on 9 % polyacrylamide gel with 35–60 % denaturing gradient, as previously described [46]. Gels were stained in 50 ml of 1× TAE with SYBR Green I dye (0.001 %) for 30 min and visualized by UV light using the Vilber Lourmat System (Marne La Vallée, France). Amplicons of interest were cut from the stained polyacrylamide gel by a sterile scalpel blade. Sterile distilled H_2_O (100 μl) was added to the excised gel fragment and subject to centrifugation at 10,000 rpm for 10 min to elute DNA. 1 μl of this solution was used for amplification with primers FP341 and RP534 under the same PCR program, as mentioned above. The PCR products were purified using QIAquick PCR purification kit (Qiagen, Hilden, Germany) and sequenced using ABI PRISM^®^ BigDye^®^ Terminator v3.1 Cycle Sequencing Kit (Applied Biosystems, Inc, Foster City, CA, USA) with a PCR thermocycler T-personal Combi (Biometra, GmbH, Goettingen, Germany). Products from sequencing were subsequently purified using BigDye purification kit (Applied Biosystems Inc) and analyzed on 3100 Avant Genetic Analyser (Applied Biosystems Inc) in the Institute of Animal Science sequencing facility (Prague, Czech Republic). The sequences were compared to those in the GenBank database using the BLASTn algorithm [47]. All sequences that did not gave meaningful result were excluded from this search.

### Scoring and analysis of bands

Scanned gels were analyzed with BioNumerics (version 7.1, Applied Maths, Sint-Martens-Latem, Beigium). Similarity indices of bands was calculated by using Pearson correlation coefficient and displayed graphically as a dendrogram [48]. The Shannon-Wiener index of diversity was used as a parameter to determine the diversity of taxa present in microbial communities sampled from cecum of HMA mice with and without DSS treatment according to Konstantinov et al. [49].

### Next generation sequencing analysis and bioinformatics

For library preparation V3 and V4 region of 16S rRNA was amplified in triplicate PCR reaction using primer pair 341F (CCTACGGGNGGCWGCAG) and 806R (GGACTACHVGGGTWTCTAAT) to utilize to the maximum read length of employed 2 × 300 pair-end sequencing at Illumina MiSeq platform (San Diego, CA, USA). Double-indexing was applied to allow for demultiplexing of output reads into original samples. Each PCR reaction was prepared in 25 µl volume using premixed mastermix (AmpliTaq Gold 360 Master Mix, Thermo Fisher Scientific, Waltham, MA, USA) and 0.8 µM of each primer following subsequent cycling conditions: initial denaturation at 95 °C for 3 min following by 35 cycles of 30 s at 94 °C, 1 min at 55 °C and 75 s at 72 °C with final extension for 10 min at 72 °C followed by hold at 4 °C. PCR reactions were checked on agarose gel for presence of expected product in samples and its absence in negative controls. Next, the triplicates from the same template reactions were pooled and cleaned using UltraClean htp 96 well PCR clean-up kit (MoBio, Carlsbad, CA, USA). Concentrations of cleaned samples were measured fluorescently with Quant-iT dsDNA Assay kit (Thermo Fisher Scientific). Sequencing adapters were ligated to the PCR amplicons with the help of TruSeq PCR-Free LT Sample preparation Kit following manufacturer instructions (Illumina, Inc). Next, sample libraries were pooled in equimolar concentration to produce final library, which was sequenced on Illumina MiSeq instrument at Genomics Core Facility, CEITEC (Brno, Czech Republic). Negative control sample (water) was run through all procedures including DNA extraction, library preparation and sequencing. Sequencing data were processed using QIIME 1.8.0 [50]. Forward and reverse reads were joined to create contigs. Afterwards reads were demultiplexed in parallel with quality filtering allowing minimal Phred quality score of Q20 and maximum number of consecutive low quality base calls of 12 due to the nature of lower quality overlaps of pair-end reads. Resulting reads were clustered to operational taxonomic units (OTUs) using UCLUST with 97 % similarity threshold against bacterial 16S reference database Greengenes gg_13_8 release [51, 52]. Singletons were discarded before producing final dataset. Taxonomic assignment of created OTUs was performed employing RDP classifier [53]. Finally information about read counts for all OTU clusters from all samples together with taxonomic information was output in OTU table. Taxa detected in negative control sample were screened out based on their relative proportional abundance. Resampling to the sequencing depth of 8000 reads per sample was performed to allow comparison of beta diversity measures. The quantitative or qualitative measures of beta diversity of samples were compared using weighted or unweighted UniFrac pairwise dissimilarity matrices, respectively [54]. To measure alpha diversity we calculated Chao1 species richness estimators [55]. Raw demultiplexed sequencing data, with sample annotations, were submitted to the Short Read Archive (http://www.ncbi.nlm.nih.gov/Traces/sra/) under the study accession number [SRP066136; http://www.ncbi.nlm.nih.gov/sra/SRP066136].

### Statistics

The differences between control group and multiple experimental groups (GF vs. all other healthy mice, DSS-treated GF mice vs. all other DSS-treated groups of mice and DSS-treated CV mice vs. all other DSS-treated mice) were analyzed with one-way analysis of variance (ANOVA) with Dunnet’s multiple comparison test. Differences between DSS-treated mice and their healthy littermates or changes in bacteria biodiversity between generations were evaluated using an unpaired two-tailed Student’s t test. Data were expressed as mean ± standard deviation (SD). Differences were considered statistically significant at P < 0.05. GraphPad Prism statistical software (version 5.03, GraphPad Software, Inc. La Jolla, CA, USA) was used for analyses.

## Availability of data and material

The dataset supporting the conclusions of this article is available in the Short Read Archive repository, [SRP066136; http://www.ncbi.nlm.nih.gov/sra/SRP066136].

## Additional file

10.1186/s13099-015-0080-2 The sequences of excised DGGE bands.

## Abbreviations

aHMA: mice associated with human microbiota from biopsy a
bHMA: mice associated with human microbiota from biopsy b
cHMA: mice associated with human microbiota from biopsy c
CCS: clinical colitis score
CD: Crohn’s disease
CV: conventional (i.e. colonized with normal commensal microbiota)
DGGE: denaturing gradient gel electrophoresis
DSS: dextran sulfate sodium
F1/3/4: 1st, 3rd and 4th filial generation
FISH: fluorescence in situ hybridization
GF: germ-free
HMA: human microbiota-associated
IBD: inflammatory bowel diseases
IFN-γ: interferon-γ
IL-10: interleukin-10
MPO: myeloperoxidase
OTUs: operational taxonomic units
PCR: polymerase chain reaction
TNF-α: tumor necrosis factor-α
UC: ulcerative colitis
